# Contemporary Practices in Refractory Out-of-Hospital Cardiac Arrest: A Narrative Review

**DOI:** 10.3390/medicina61061053

**Published:** 2025-06-07

**Authors:** Jan Jezeršek, Matej Strnad

**Affiliations:** 1 Community Health Centre Dr. Adolfa Drolca Maribor, 2000 Maribor, Slovenia; matej.strnad@um.si; 2 Faculty of Medicine, University of Maribor, 2000 Maribor, Slovenia; 3 University Medical Centre Maribor, 2000 Maribor, Slovenia

**Keywords:** ECMO, out-of-hospital cardiac arrest, double-sequential defibrillation, beta-adrenergic receptor blockade

## Abstract

Out-of-hospital cardiac arrest remains a major cause of adult mortality worldwide, with survival to hospital discharge rates around 10%. Despite advances in prehospital care, rapid recognition and high-quality chest compressions are the primary interventions, while early defibrillation is one of the few measures shown to improve survival. This literature review examines novel interventions for patients with refractory ventricular fibrillation and pulseless ventricular tachycardia, focusing on double sequential defibrillation, beta-adrenergic receptor antagonists, and extracorporeal cardiopulmonary resuscitation. Evidence suggests that double sequential defibrillation may improve survival to discharge in refractory ventricular fibrillation, but consensus and large-scale validation are lacking. Beta-blockers show promise for increasing the rates of return of spontaneous circulation and favourable neurological outcomes, yet robust evidence is still needed. Extracorporeal cardiopulmonary resuscitation, particularly when initiated rapidly in selected patients, can enhance survival and neurological outcomes, though studies show mixed results and highlight the importance of patient selection and system readiness. Overall, while these interventions offer potential, their widespread adoption requires further high-quality research to determine efficacy, optimal protocols, and resource implications in both prehospital and emergency department settings.

## 1. Introduction

Out-of-hospital cardiac arrest (OHCA) represents a considerable cause of mortality among the adult population, affecting some 4 million people globally per annum. The overall survival rate to hospital discharge is approximately 10% [1,2,3,4]. The annual incidence in Europe ranges from 19 to 97 cases per 100,000 inhabitants [3,5]. Advancements in the field of scientific research have precipitated enhanced prehospital management strategies, which have subsequently resulted in an increased rate of hospital admissions [5,6]. Despite the introduction of novel interventions, the rapid recognition of cardiac arrest and the prompt initiation of high-quality chest compressions remain the mainstay of management [1,5,7,8,9]. There are four electrophysiologic entities that can present as a cardiac arrest, known as the arrest rhythms. These are asystole, pulseless electrical activity (PEA), pulseless ventricular tachycardia (pVT), and ventricular fibrillation (VF). The first two are non-shockable and carry a poorer prognosis (estimated at below 10%), as no causative treatment is readily available. Roughly 20–30% of OHCAs have an initial shockable rhythm (pVT and VF) and are associated with a higher survival (estimated at around 30%) due to the curative potential of early defibrillation, one of the few interventions known to favourably impact survival rates [1,7,9]. The standard advanced cardiac life support (ACLS) protocol in these rhythms includes increasing the energy levels of the biphasic defibrillator (200–300–360 J) in coalition with the following pharmaceutical therapy: epinephrine at 1 mg after the third shock (and then every 3–5 min) and amiodarone (300 mg after the third and 150 mg after the fifth shock) [2,6,7]. Studies have demonstrated the efficacy of double sequential defibrillation in cases of refractory ventricular fibrillation concerning survival to discharge [2,7,10,11,12]. A similar conclusion has been reached in the context of targeted beta-adrenergic receptor blocker use, such as esmolol. Most studies found that it may increase the rates of return of spontaneous circulation (ROSC), survival to intensive care unit (ICU), and favourable neurologic outcomes; however, more robust evidence is needed to determine the extent of its uses [1,7,13,14,15]. Recent findings have demonstrated the efficacy of extracorporeal cardiopulmonary resuscitation (ECPR) in enhancing both survival and favourable neurological outcomes, particularly in the cases of witnessed cardiac arrest with a reversible cause. The effectiveness of ECPR is contingent upon the prompt initiation of high-quality chest compressions, the presence of an initial shockable rhythm, and a relatively brief emergency medical services (EMS) response time [7]. The purpose of this paper is to review the relevant scientific literature on novel interventions both in the prehospital setting and in the emergency department in OHCAs with refractory VF and pVT, namely the use of double sequential defibrillation, the administration of beta-adrenergic receptor antagonists, and extracorporeal CPR (both in and out of hospital).

## 2. Double Sequential Defibrillation (DSD)

Double sequential defibrillation is an alternative defibrillation strategy used in patients with refractory VF, utilizing two defibrillators in quick succession to deliver two transthoracic shocks [10,16]. Early defibrillation is crucial in cardiac arrest patients with a shockable rhythm (e.g., VF or pVT) [5,7,10]. These account for approximately one-third of OHCAs and represent a subgroup of patients in which survival with favourable neurologic outcome is the greatest (reported at approx. 30%) in comparison to non-shockable rhythm OHCAs (e.g., PEA and asystole) [6,11,12]. Within this group, a small fraction (estimated at around 0.6 cases per 100,000 inhabitants) of patients remain in arrhythmia (refractory VF, pVT) despite initiating standard ACLS protocol [6,10,15]. The diagnosis of refractory VF can be confirmed after at least three unsuccessful defibrillation attempts and the administration of 3 mg of adrenaline and 300 mg of amiodarone [6,12,14,15,17]. It has been shown that the vast majority of these patients (70–85%) have concomitant coronary artery disease, rendering standard ACLS resuscitation ineffective [18]. The survival of these patients is significantly lower (4.9–12.7%) compared to the survival of patients with recurrent VF/pVT (21.4–29.3%) [6,11,12,14,15]. There are no efficacious treatments that could benefit patients in VF/pVT refractory to standard defibrillation [6].

The origins of double sequential defibrillation can be traced back to the 1980s, a period marked by significant advancements in the field of defibrillation research. During this time, it was observed that sequential shocks reduced the total energy requirement for successful defibrillation [6]. These studies led to the formulation of two theories. The first, known as the threshold theory, posits that initial defibrillation renders cardiomyocytes more susceptible to subsequent defibrillation [6,19]. The second, termed the vector theory, suggests that altering the vector of the electrical impulse can enhance the probability of successful defibrillation [6,10,11,19]. In clinical practice, these two theories are often integrated through the implementation of two pairs of defibrillation pads, which are positioned on the patient. One pair is placed anterolaterally, while the other is positioned anteroposteriorly or adjacent to the first [6,19,20]. This approach aims to enhance the cardiomyocyte response, given their directional sensitivity. The spatiotemporal displacement of electrical currents ensues as neither shock is delivered at precisely the same moment [6,10,20]. Once DSD or vector change (VC) defibrillation has been initiated (after three unsuccessful standard defibrillation attempts), it is utilized until the end of reanimation. The delay between both shocks is crucial for safety, as simultaneous shocks were reported to damage the defibrillators, but should not exceed 1 s [11,16].

DSD was shown to be superior to standard defibrillation for survival to hospital discharge by most studies, but expert consensus has not yet been achieved [2,6,7,10,11,12,20,21]. Miraglia et al. found, in their 2020 scoping review, that there is a lack of literature and evidence to support the large-scale use of DSD [6]. A multicentre, retrospective cohort study conducted in 2023 by Stupca et al. found that the combination of esmolol administration, VC defibrillation, and epinephrine restriction resulted in decreased rates of ROSC in comparison to standard ACLS (19.1% and 66.7%, respectively), while the differences in neurologically intact survival at discharge and survival to discharge were not statistically significant [21], whereas a pilot randomized control trial (RCT) performed by Cheskes et al. in 2020, implementing the DOuble SEquential external defibrillation for refractory ventricular fibrillation (DOSE VF) protocol found that vector change and DSD resulted in increased rates of ROSC in comparison to standard defibrillation (39.3% for VC, 40.0% for DSD, and 25.0% for standard defibrillation) and were more successful at VF termination (82.0% for VC, 76.3% for DSD, and 66.6% for standard defibrillation) [11]. A cluster RCT published in 2022 by Cheskes et al. found that DSD was superior to standard defibrillation at achieving ROSC (46.4% and 26.5%, respectively), survival to hospital discharge (30.4% and 13.3%, respectively), and the termination of VF (84.0% and 67.6%, respectively) [16]. The same group confirmed their pilot study results in a 2024 secondary analysis study, highlighting the beneficial effects of DSD on survival to discharge compared to standard defibrillation (27.2% and 14.0%, respectively) [12]. A separate secondary analysis study of DOSE VF RCT by Deb et al. (2024) reported that patients treated with DSD had a higher chance of survival to hospital admission in comparison to non-DSD patients (*p* = 0.02) while the data for survival to hospital discharge and neurologically intact survival were not statistically significant (*p* = 0.16 and *p* = 0.15, respectively) [2]. Recent European Resuscitation Council (ERC) (2021) guidelines do not yet recommend the routine use of DSD; they do, however, suggest using alternative pad placement, i.e., vector change defibrillation, in refractory VF [20].

In conclusion, there is a lack of high-quality evidence supporting the standard use of alternative defibrillation strategies. In the reviewed literature, substantial differences in protocols were noticeable. Further evidence is needed to establish a uniform standard considering pad placements, defibrillation settings, inter-shock delay, the number of operators, etc. However, despite requiring two defibrillators, at least one skilled operator, and improved peri-patient surroundings management, DSD is a promising solution for a distinct subset of patients.

## 3. Beta-Adrenergic Receptor Antagonist Infusion

Beta-blockers, particularly esmolol, have emerged as a potential adjunctive therapy for treating refractory VF or pVT during OHCA [7,13,17,19,22]. These conditions are characterized by their resistance to standard ACLS interventions, such as defibrillation and epinephrine administration [13,15,17,20]. Beta-adrenergic receptor blockers may counteract the adverse beta-adrenergic effects of epinephrine, including increased myocardial oxygen demand and heightened arrhythmogenicity, particularly in high doses [13,14,15,17,22]. Esmolol, an ultra-short-acting beta-1 selective antagonist, has shown promise due to its rapid onset and short half-life, reducing the risk of prolonged adverse effects [14,15,22]. However, it has been studied primarily through observational studies and case reports with considerable selective bias. The positive effects of esmolol stem from its pharmacodynamic properties. The competitive blockade of beta-1 receptors in the myocardium produces negative chrono-, ino-, and dromotropic effects, essentially decreasing arrhythmogenicity, lowering the cardiac workload, and improving the defibrillation success rate, therefore decreasing the deleterious effects of high-dose epinephrine, while maintaining positive alpha-adrenergic receptor activity in the periphery (i.e., vasoconstriction) [14,19]. In the reviewed literature, esmolol was initiated after three unsuccessful defibrillation attempts with a loading dose of 500 mcg/kg, succeeded by a continuous infusion of 0–100 mcg/kg/min [14,15,17]. In this aspect, two antiarrhythmics were also studied: lidocaine, a class Ib antiarrhythmic that blocks fast sodium receptors in non-nodal tissue, and amiodarone, a class III antiarrhythmic that blocks potassium rectifier currents responsible for the repolarization of the myocardium. Either can be used in witnessed OHCA with refractory VF as a part of standard ACLS protocol [19,22].

A 2021 literature review by Roach et al. found that the use of esmolol during resuscitation in refractory VF OHCA may mitigate the deleterious effects of epinephrine in comparison to standard ACLS, improving the rates of ROSC (67.0% and 32.0%, respectively) and survival to discharge with good neurologic function (50.0% and 11.0%, respectively) [23]. In 2019, Hwang et al. were the first to publish a case report of a 51-year-old male with a refractory VF ST-elevation myocardial infarction (STEMI) that responded to low-dose esmolol administration [22]. A small single-centre retrospective case–control study conducted on 41 patients by Lee et al. in 2016 reported that the administration of esmolol may increase the rates of sustained ROSC in patients with refractory VF in comparison to the non-esmolol group (56.0% and 16.0%, respectively; *p* = 0.007) [14]. Likewise, a systematic review and meta-analysis by Gottlieb et al. from 2020 found that a beta-adrenergic receptor blockade may, in comparison to standard procedure, improve both of the rates of sustained ROSC (59.1% and 22.7%, respectively), survival to discharge (53.1% and 10.6%, respectively), survival to admission (59.1% and 22.7%, respectively), and rates of a favourable neurologic outcome (27.3% and 9.1%, respectively) [13]. The same year, Miraglia et al. published their systematic review and meta-analysis, which found that evidence for the routine use of esmolol during resuscitation was inconclusive, highly confounding, and may even be harmful [15]. A 2022 observational analysis by Patrick et al. found that the routine use of esmolol in the prehospital setting is feasible and is non-inferior to standard ACLS. They reported higher rates of ROSC, without statistical significance [17]. A recent multicentre, retrospective cohort study by Stupca et al. (2023) found that the combination of esmolol administration, vector change defibrillation, and epinephrine restriction resulted in decreased rates of ROSC in comparison to standard ACLS (19.1% and 66.7%, respectively), while the differences in neurologically intact survival at discharge and survival to discharge were not statistically significant [21]. In contemporary ERC guidelines, the beta-adrenergic receptor blockade is not mentioned in the context of refractory ventricular arrhythmias [20].

Despite its promise, esmolol remains an experimental therapy for refractory VF. Current evidence does not establish definitive benefits for long-term survival or neurological recovery. Randomized controlled trials are urgently needed to validate its efficacy and safety, optimize dosing protocols, and clarify its role within ACLS guidelines [13,14,15,17,22,23]. Until then, esmolol should be considered a potential but unproven adjunct.

## 4. Extracorporeal Cardiopulmonary Resuscitation (ECPR)

ECPR is an advanced resuscitation technique that incorporates veno-arterial extracorporeal membrane oxygenation (V-A ECMO) into traditional cardiopulmonary resuscitation (CPR) [24,25,26,27]. In V-A ECMO, deoxygenated venous blood is drained by way of a cannula from the right atrium through an external oxygenator, which regulates O_2_ and CO_2_ levels. The oxygenated arterial blood is returned into the proximal aorta by a return cannula to complete the circuit [28,29]. ECPR provides temporary mechanical support for both cardiac and pulmonary functions, ensuring oxygenated blood flow to vital organs while the underlying causes of cardiac arrest are addressed [7,24,25,26].

Studies indicate that ECPR can improve survival rates in carefully selected patients, particularly those aged <65, with a witnessed cardiac arrest with well-performed CPR, an initial shockable rhythm, a refractory cardiac arrest with a reversible cause, a quick EMS response time, shorter durations of CPR before ECMO initiation (in many protocols, not more than 60 min of ongoing CPR), and patients that had ECMO implanted in the prehospital setting, which is rare [7,18,25,30,31]. The studies reviewed by this article include different combinations of the above-mentioned factors as inclusion criteria, further complicating inter-study comparison. The most common inclusion criteria are shown in Table 1.

Several studies have evaluated the efficacy of ECPR in OHCA. The first RCT to show that ECPR improves survival compared to standard CPR was a single-centre (University of Minnesota) RCT, published in 2020 by Yannopoulos et al., termed the ARREST trial [7,18]. It demonstrated that early ECMO-facilitated resuscitation significantly improved survival to hospital discharge (42.9% vs. 6.7%) compared to standard ACLS in patients with refractory VF who were randomized to one of the protocols [18]. Bartos et al. highlighted the importance of time-to-ECMO as an independent predictor of survival in their single-centre (University of Minnesota) retrospective study of adult OHCAs with VF/pVT, published in 2020 [32], whereas a pivotal prospective registry study involving 13,191 OHCAs in Paris published the same year by Bougouin et al. found no significant difference in survival to hospital discharge between ECPR and conventional CPR (8.0% vs. 9.0%, respectively) but highlighted that early ECMO initiation and factors like initial shockable rhythm and ROSC improved outcomes [25]. Likewise, a single-centre RCT (Prague OHCA study) by Belohlavek et al. in 2022 found that an invasive strategy, comprising intra-arrest transport, early coronary angiography, and ECPR, found no statistically significant improvement in neurologically favourable outcome at 180 days compared to standard ACLS protocol; however, a notable difference in favour of the invasive strategy was highlighted (31.5% vs. 22.0%, respectively) [33]. A secondary analysis of this trial conducted by Rob et al. in 2022 found that ECPR was associated with improved survival at day 180 in patients without ROSC. Factors like initial shockable rhythm and younger age were also found to be predictive of improved survival [34]. A recent meta-analysis of four RCTs by Scquizzato et al. in 2023 compared survival with favourable neurologic outcome at the longest follow-up available and found that ECPR outperformed conventional CPR (34% vs. 23%, *p* = 0.01) [35]. A 2024 meta-analysis of three RCTs by Heuts et al. looked at neurologically favourable survival at 6 months and found a 75.8% posterior probability of a clinically relevant ECPR treatment effect (>5%) [36].

As time-to-ECMO was found to be an important independent predictor of survival [4,9,32,37,38], multiple studies evaluated the efficacy and feasibility of prehospital ECPR. The first study to evaluate the use of prehospital ECPR by an emergency physician and/or intensive care physician was conducted in 2013 by a Paris group led by Lamhaut et al. They found that prehospital ECPR is safe and feasible in observed settings. However, the study was not designed to assess survival [39]. In 2017, Lamhaut et al. published a publicly reverberating case report of an ECPR in a prehospital setting (Louvre museum in Paris), highlighting its advantages, namely reducing time-to-ECMO in patients with a difficult extraction, and disadvantages, namely that it necessitates a highly trained team and presents an organizational challenge [40]. A review article by Singer et al., published in 2018, found a lack of standardized inclusion criteria and concluded that further evidence is needed to justify extensive resource utilization [41]. A registry study by Bougouin et al. published in 2020 compared hospital and prehospital ECPR. The latter was an independent predictor of higher hospital survival (*p* = 0.006) and favourable neurologic outcomes (*p* = 0.008) [25]. Likewise, in 2022, in a letter to the editor, Pozzi et al. presented their findings. After implementing a prehospital ECPR strategy, they recorded an increase in survival with neurologically favourable outcomes (23.5% vs. 33.3%, respectively) in OHCA patients with a refractory VF [42]. In 2023, a CHEER3 prospective feasibility study by Richardson et al. was published. It showed promising results, with a 40.0% neurological intact survival, while using similar inclusion criteria to the preceding studies, improving inter-study comparability [30]. Similar results were published by Bartos et al. in 2020, describing the Minnesota mobile ECPR consortium, reaching rates of neurologically favourable survival comparable to prior studies (43.0%) [38]. An editorial by Scquizzato et al. in 2023 highlighted the advantages of prehospital ECPR, namely a reduction in low-flow time, increasing the yield of eligible patients and the equity of access, and its disadvantages, namely the requirement of specialized equipment and skills, complex procedural work, and diagnostic and treatment delays. The article concluded that the evidence supporting prehospital ECPR is promising but of very low certainty [31]. Singer et al. published a prospective, open-label, feasibility study, Sub30, in 2024, demonstrating that prehospital ECPR was safe, timely, and effective [43]. The ON-SCENE RCT by a Netherlands group is underway; however, the study will not be completed until 2026.

Experimental models have shown that ischemia–reperfusion injury is detrimental to the function of the brain as the most sensitive organ to ischemia [44]. To alleviate this effect, a physiologically appropriate, cardiac surgery-based technique, termed the controlled automated reperfusion of the whole body (CARL), was developed [44,45,46]. It comprises four cardinal advantages:

- Dual-pump ECMO, enabling pulsatile flow and controlled oxygenation;
- Adjustment of 14 blood parameters (e.g., electrolyte disbalance, osmolarity, oxygenation, free radical scavengers, etc.);
- Comprehensive real-time monitoring (cardiac output, heart rate, invasive blood pressure, blood gas, and electrolyte analysis);
- Out-of-hospital CARL option [45].

In a case series published by Philipp et al. in 2023, the application of CARL was found to be beneficial for the following reasons: invasive arterial pressure monitoring, oxygen saturation control, and built-in blood gas analysis [46]. A multicentre, prospective observational study published in 2024 by Trummer et al. highlighted that the use of CARL has the potential to further improve outcomes and restore renal, hepatic, and pulmonary functions, resulting in a higher survival rate in prehospital cannulation as opposed to in-hospital cannulation (57.1% and 35.0%, respectively); however, further studies are required to define clinical relevance [45].

In summary, ECPR represents a transformative approach to managing refractory cardiac arrest, but it is highly invasive, potentially causing dangerous adverse effects, such as puncture site hematomas, and requires numerous well-equipped, highly specialized professional team members. It poses an organizational and fiscal challenge even for highly developed healthcare systems. Its use is justified in select cases in well-resourced tertiary systems and research centres with an expansive background of supporting services and professions [7,18,30,31,33,42,47]. While evidence generally supports its lifesaving potential and long-term neurologic benefits in selected scenarios, results are contradictory, and further research is needed to refine protocols and enhance accessibility [3,7,30,31,32,42,47].

All therapeutic modalities that are in the scope of this article are presented concisely in Table 2, as shown below.

## 5. Conclusions

In conclusion, the novel practices in refractory OHCAs reviewed by this article have shown promise in limited studies and selected patients. Few multicentred RCTs have been conducted. Results are often contradictory or confounding. Some well-designed studies are still underway, and their results might cause a paradigm shift in the approach to refractory OHCA. Until then, however, DSD, beta-adrenergic receptor antagonists, and ECPR remain methods for use in carefully selected cases and well-resourced research settings.

## Figures and Tables

**Table 1:** ECPR initiation criteria.

Criterion	Typical Threshold/Requirement
Age	<65 years
Witnessed arrest	Yes, with immediate (<5 min) high-quality CPR
Initial rhythm	Shockable rhythm (VF/pVT)
ROSC	Present
Low-flow time (arrest-to-ECPR)	<60 min
Cause	Reversible cardiac

**Table 2:** Review of novel approaches in refractory OHCA.

Underlying MoA	Target Organ System	Agent		Explanation	Reference
**Electrophysiological**	Cardiac	DSD		Higher energy overcomes the defibrillation threshold (threshold theory). The first shock primes cardiomyocytes, rendering them susceptible to subsequent shock. Vector theory (see below).	[6,16,19]
		VC		Altering the vector of electrical current increases the probability of successful defibrillation (vector theory).	[10,11,16,19]
**Pharmaceutical**	Cardiac	Beta-adrenergic receptor blockade	Esmolol	Counteracting the adverse beta-adrenergic effects of epinephrine, which include increased myocardial oxygen demand and heightened arrhythmogenicity, which take place particularly in high doses.	[13,15,17,19,22]
**Mechanical**	Cardiopulmonary	ECPR	ECMO	Provides temporary mechanical support for both cardiac and pulmonary functions, ensuring oxygenated blood flow to vital organs while the underlying causes of cardiac arrest are addressed.	[7,24,25,26]
			CARL	Pulsatile flow, blood parameter control, and real-time monitoring simulate physiologic conditions, decreasing the deleterious effects of ischemia–reperfusion injury to the brain.	[44,45,46]

## Abbreviations

The following abbreviations are used in this manuscript:

OHCA	Out-of-hospital cardiac arrest
PEA	Pulseless electrical activity
pVT	Pulseless ventricular tachycardia
VF	Ventricular fibrillation
ROSC	Return of spontaneous circulation
ICU	Intensive care unit
ECPR	Extracorporeal cardiopulmonary resuscitation
EMS	Emergency medical services
DSD	Double sequential defibrillation
ACLS	Advanced cardiac life support
RCT	Randomized control trial
DOSE VF	DOuble SEquential external defibrillation for refractory Ventricular Fibrillation
VC	Vector change
ERC	European Resuscitation Council
STEMI	ST-elevation myocardial infarction
V-A ECMO	Veno-arterial extracorporeal membrane oxygenation
CPR	Cardiopulmonary resuscitation
CARL	Controlled automated reperfusion of the whole body
MoA	Mechanism of action
